# The Curability of Hysteria

**Published:** 1868-12

**Authors:** R. L. Walston

**Affiliations:** Paris, Ill.


ARTICLE XLIX.

### THE CURABILITY OF HYSTERIA.

By R. L. WALSTON, M.D., Paris, Ill.

The teaching of the advance of the medical profession upon the subject of hysteria is, that it is a manifestation, through the nervous system, of structural lesions, of physical changes, of absolute local disease, amenable to treatment and to cure. My little experience corroborates, yea, demonstrates, the correctness of the above teaching. And, for the purpose of illustrating, I hereby report in brief what I consider a typical case.

In May, 1867, while treating a surgical case of great interest, my attention was called to a woman doing kitchen service in the house of my patient. There were present all the symptoms of this protean malady, and not the least trifling of which was the enormous distention of the bladder. Extract of valerian, morphia, and the catheter disposed of the case, and I saw little else of it till in June, 1868. The patient was proverbially hysterical, and had been treated by all the physicians in this place, and some from abroad; but I believe all (myself among the rest) had aimed at palliation only. This time I determined to ascertain, and, if possible, remove the cause of my patient's suffering.

A careful examination revealed hyperæsthesia of all the pelvic organs, with some traces of cystitis and cervitis. I had almost neglected to state, that this patient had suffered for eight years with distressing dysmenorrhœa, and for two years just past had been menstruating vicariously, the stomach having taken on the office of the uterus. Some of her answers led me to suspect disease of the bladder, produced by physical cause; and, upon sounding the bladder, I discovered a calculus, which was speedily removed.

The stone was of a pale-yellow color, ovoid in shape, flinty in texture, with calcareous concretions upon it sufficient to cover about half of the surface, serving to make it rough, and capable of a vast amount of irritation; and had evidently been introduced through the urethra by the patient herself, some ten years or more ago.

I now have the pleasure to report her *perfectly* well, cured of her hysteria (?), and menstruating regularly and naturally.

REMARKS.—This case is valuable only so far as it serves to impress upon the minds of the profession that hysterical manifestation is the result of local causes, which can usually be found about the uterine organs; and so long as caustics, properly applied, will cure those chronic inflammations, and sponge-tents, knives, and écrasseurs remove polypi, fibrous and fibroid tumors, I, for one, shall use them, to the joy and satisfaction of myself, and the cure of my patients.

